# Red Yeast Rice Protects Circulating Bone Marrow-Derived Proangiogenic Cells against High-Glucose-Induced Senescence and Oxidative Stress: The Role of Heme Oxygenase-1

**DOI:** 10.1155/2017/3831750

**Published:** 2017-05-06

**Authors:** Jung-Tung Liu, Huey-Yi Chen, Wen-Chi Chen, Kee-Ming Man, Yung-Hsiang Chen

**Affiliations:** ^1^School of Medicine, College of Medicine, Chung Shan Medical University, Taichung 402, Taiwan; ^2^Department of Neurosurgery, Chung Shan Medical University Hospital, Taichung 402, Taiwan; ^3^Graduate Institute of Integrated Medicine, College of Chinese Medicine, Research Center for Chinese Medicine & Acupuncture, China Medical University, Taichung 404, Taiwan; ^4^Departments of Obstetrics and Gynecology, Urology, Anesthesiology, and Medical Research, China Medical University Hospital, Taichung 404, Taiwan; ^5^Department of Medicinal Botanicals and Health Applications, Da-Yeh University, Changhua 515, Taiwan; ^6^Department of Psychology, College of Medical and Health Science, Asia University, Taichung 413, Taiwan

## Abstract

The inflammation and oxidative stress of bone marrow-derived proangiogenic cells (PACs), also named endothelial progenitor cells, triggered by hyperglycemia contributes significantly to vascular dysfunction. There is supporting evidence that the consumption of red yeast rice (RYR; *Monascus purpureus*-fermented rice) reduces the vascular complications of diabetes; however, the underlying mechanism remains unclear. This study aimed to elucidate the effects of RYR extract in PACs, focusing particularly on the role of a potent antioxidative enzyme, heme oxygenase-1 (HO-1). We found that treatment with RYR extract induced nuclear factor erythroid-2-related factor nuclear translocation and HO-1 mRNA and protein levels in PACs. RYR extract inhibited high-glucose-induced (30 mM) PAC senescence and the development of reactive oxygen species (ROS) in a dose-dependent manner. The HO-1 inducer cobalt protoporphyrin IX also decreased high-glucose-induced cell senescence and oxidative stress, whereas the HO-1 enzyme inhibitor zinc protoporphyrin IX and HO-1 small interfering RNA significantly reversed RYR extract-caused inhibition of senescence and reduction of oxidative stress in high-glucose-treated PACs. These results suggest that RYR extract serves as alternative and complementary medicine in the treatment of these diseases, by inducing HO-1, thereby decreasing the vascular complications of diabetes.

## 1. Introduction

Endothelial dysfunction-related atherosclerosis is typically multifactorial. It is most often dependent on inflammatory risk factors such as hyperglycemia, hypercholesterolemia, hypertension, smoking, and obesity [1, 2]. Complications from atherosclerotic-related diseases remain the leading cause of mortality and morbidity in various industrialized countries [3].

Hyperglycemia, which is associated with endothelial dysfunction, is a primary cause of vascular complications in diabetes [4]. Evidence shows that the repair of endothelium involves bone marrow-derived proangiogenic cells (PACs), also known as endothelial progenitor cells (EPCs), in vasculogenesis [5]. The impaired function and reduced number of EPCs were found to be associated with vascular complications in both type I and type II diabetes [6, 7]. In addition, our previous studies have shown that hyperglycemia directly impairs the biological functions of angiogenesis, induces cellular aging (senescence), and produces reactive oxygen species (ROS) in EPCs [8–11].

The definition of EPCs has changed over the years as many studies have revealed the true face of the majority of EPC heterogeneity, which are in fact not endothelial precursors but can be described as myeloid-lineage-derived cells with proangiogenic properties. EPCs were classically described as cells that expressed a combination of endothelial and progenitor markers; however, none of these markers is fully specific [12–14]. Thus, other names, such as bone-marrow-derived PACs, have been suggested for EPCs. Nevertheless, despite their history and controversy, EPCs have been applied to different cell types that play roles in the regeneration of the endothelial lining in vasculature. EPCs in all their forms remain a promising target of regenerative medicine.

Red yeast rice (RYR; *Monascus purpureus* Went-fermented rice) has been used for many centuries to make rice wine in China, to maintain food taste and color, and for its medicinal properties. Biological and epidemiological evidence support that the intake of RYR may reduce the incidence of atherosclerosis. RYR contains naturally occurring statins that have serum lipid-modulating effects. Thus, RYR has a lipid-lowering effect in subjects with hyperlipidemia. Pharmacological RYR-related products are marketed in China, Taiwan, and in the United States. RYR has also been shown to have free radical scavenging abilities and can protect the function of endothelium through antioxidative and anti-inflammatory mechanisms [15]. Moreover, a previous study showed that RYR inhibited homocysteine-induced endothelial adhesion via intracellular ROS reduction [16].

Heme oxygenase-1 (HO-1) is a member of the heat shock protein family. The expression of HO-1 is triggered by various stressors, including oxidative stress, heavy metals, UV radiation, and hypoxia [17]. HO-1 expression is mediated through accumulation of the nuclear factor erythroid-2-related factor (Nrf2) in the nucleus [18]. HO-1 was found to be a pivotal antioxidative, anti-inflammatory, and antiapoptotic molecule [19]. Various medicinal plant-derived chemical substances may induce HO-1 activation and can maximize the intrinsic antioxidative abilities [20].

In this study, we explored the potency of RYR extract as an HO-1 inducer in PACs and investigated whether it contributed to the beneficial effects against PAC senescence and oxidative stress.

## 2. Materials and Methods

### 2.1. Materials

Glucose, mannitol, and other chemicals were obtained from Sigma Chemical Co. (MO, USA). RYR (LipoCol Forte) was obtained from NatureWise Biotech & Medicals Corporation (Taipei, Taiwan) and extracted at room temperature [15, 16]. Final concentration of solvents in following studies was always less than 0.5% to avoid potential interference. The cobalt protoporphyrin IX (CoPPIX) and zinc protoporphyrin IX (ZnPPIX) used (10 *μ*M) did not significantly influence cell viability (>90%).

### 2.2. PAC Isolation, Cultivation, and Identification

The protocol conforms to the Helsinki declaration. China Medical University (Taichung, Taiwan) Institutional Review Board approved the study by expedited review. Peripheral blood mononuclear cells (MNCs) were isolated (gradient centrifugation) from volunteers by Histopaq-1077 (Sigma, USA). Isolated MNCs were plated in endothelial growth medium (EGM-2 MV; Cambrex, USA), with supplements (hydrocortisone, R^3^-insulin-like growth factor 1, human epidermal growth factor, VEGF, human fibroblast growth factor, gentamicin, amphotericin B, vitamin C, and 20% fetal bovine serum) on fibronectin-coated plates. After culturing, medium was replaced and nonadherent cells were removed. Culture medium was changed every 3 days, and a number of cells can continue to grow into late outgrowth cells. Late outgrowth PACs under passage 3 were used for the study [8].

PACs were further characterized by immunofluorescence for CD34, kinase insert domain receptor (KDR, also named vascular endothelial growth factor receptor 2), and CD31 (platelet endothelial cell adhesion molecule; PECAM-1) (Santa Cruz, USA) expressions [8].

### 2.3. Western Blot Analysis

Cell lysates were prepared in lysis buffer (20 mM Tris-HCl, 150 mM NaCl, 1 mM ethylenediaminetetraacetic acid, 1 mM ethylene glycol tetraacetic acid, 1% Triton, 2.5 mM sodium pyrophosphate, 1 mM glycerophosphate, 1 mM Na_3_VO_4_, 1 *μ*g/ml leupeptin, and 1 mM phenylmethylsulfonyl fluoride, pH 7.5). The concentration of protein was determined by the Bio-Rad Protein Assay reagent.

Nuclear protein extracts were prepared as previously described [8]. In brief, after being washed with ice-cold PBS, cells were scraped off the plates with a cell scraper in 1 mL of ice-cold buffer A (10 mmol/l HEPES/NaOH, pH 7.9; 10 mmol/l KCl; 1.5 mmol/l MgCl_2_; 1 mmol/l DTT; l0.5 mmol/l PMSF; 2 *μ*g/ml aprotinin; 2 *μ*g/ml pepstatin; and 2 *μ*g/ml leupeptin). After centrifugation at 300*g* for 10 minutes at 4°C, cells were resuspended in 80 *μ*l of buffer B (buffer A containing 0.1% Triton X-100) by gentle pipetting. Cell lysates were allowed to stand on ice for 10 minutes and then centrifuged at 12,000*g* for 10 minutes at 4°C. Nuclear pellets were resuspended in 70 *μ*L of ice-cold buffer C (20 mmol/l HEPES/NaOH, pH 7.9; 1.5 mmol/ MgCl_2_; 1 mmol/l DTT; 0.2 mmol/l EDTA; 420 mmol/l NaCl; 25% glycerol; 0.5 mmol/l PMSF; 2 *μ*g/ml aprotinin; 2 *μ*g/ml pepstatin; and 2 *μ*g/ml leupeptin), incubated on ice for 30 minutes with intermittent mixing, and then centrifuged at 15,000*g* for 30 minutes at 4°C. Nuclear protein extracts prepared as described above were determined by protein assay.

Proteins were separated by sodium dodecyl sulfate-polyacrylamide gel electrophoresis and transferred to polyvinylidene fluoride membrane. The membranes were probed with goat anti-HO-1 antibody (R&D Systems, MN, USA) or rabbit anti-Nrf2 antibody (Abcam, Cambridge, MA, USA) and then incubated with horseradish peroxidase-conjugated secondary antibodies, and the proteins were visualized with a chemiluminescence detection kit (Amersham Biosciences, NJ, USA). Mouse anti-*β*-actin (Labvision/NeoMarkers, CA, USA) or anti-lamin B (Abcam, Cambridge, MA, USA) antibodies were used as loading controls. Protein expression levels were quantified using ImageQuant (USA) software [20].

### 2.4. PAC Viability

The 3-(4,5-dimethylthiazol-2-yl)-2,5-diphenyl tetrazolium bromide (MTT, Sigma, USA) assay was used for cell viability. Briefly, PACs were grown in plates and incubated with various concentrations of agents. Medium containing MTT (0.5 mg/ml) was added. Finally, dimethyl sulfoxide was added to each well and the absorbance of blue formazan read at 540 nm using a microplate reader (Multiskan Ex, Thermo Lab systems, USA). Cells incubated in control medium were considered 100% viable [8].

### 2.5. PAC Senescence Assay

The senescence of PACs was determined by the senescent cell staining kit (Sigma, USA). Briefly, PACs were fixed for 6 min in 2% formaldehyde and 0.2% glutaraldehyde in phosphate-buffered saline and then incubated for 12 h at 37°C with fresh X-gal staining solution (1 mg/ml X-gal, 5 mM potassium ferricyanide, 5 mM potassium ferrocyanide, and 2 mM MgCl_2_; pH 6). Then, blue-stained and total cells were counted for calculating the *β*-galactosidase-positive cell percentage [8].

### 2.6. ROS Production

Effect of RYR extract on ROS production was determined by a fluorometric assay by probe dichloro-dihydro-fluorescein diacetate (DCFH-DA). Confluent cells in 48-well plates were pretreated with RYR extract. HBSS containing 10 *μ*M DCFH-DA was added, and the cells were incubated with it for 30 min. The relative fluorescence unit was measured at 485 nm excitation and 530 nm emission by a fluorescence microplate reader [21].

### 2.7. Measurement of Glutathione (GSH), Glutathione Reductase (GR), and Thiobarbituric Acid Reactive Substances (TBARS)

GSH levels were measured by a colorimetric assay (Bioxytech GSH-400; OxisResearch, Portland, OR, USA). Metaphosphoric acid (5%) was added to the cells and then scraped off it. The mixture was centrifuged at 3000*g* for 5 min at 4°C, and the supernatant was measured at 400 nm after a chemical reaction with reagent R1 (4-chloro-1-methyl-7-trifluromethyl-quinolinium methylsulfate) and reagent R2 (30% NaOH) with a GSH standard curve [22].

The GR activity was determined with a protocol described previously [23]. In brief, GR activity was expressed as a rate of decrease in absorbance at 340 nm/min due to the NADPH oxidation by GR, and the enzyme activity was normalized with mg protein.

Lipid peroxidation was quantified by TBARS determination by spectrophotometric assay (Beckman Coulter, DU 640 spectrophotometer, Germany). The lipid peroxide levels, expressed as nmol malondialdehyde/mg protein, were calculated from the absorbance at 532 nm by external standard tetraethoxypropane [22].

### 2.8. RNA Extraction and Real-Time PCR

Total RNA was isolated from lung cancer tissues and adjacent normal lung tissues of the NSCLC patients and, subsequently, analyzed by real-time PCR. The following primers were designed using Primer Express software (RealQuant, Roche) based on published sequences: human HO-1 sense primer: 5′-TTC TTC ACC TTC CCC AAC TA-3′; HO-1 antisense primer 5′-GCA TAA AGC CCT ACA GCA AC-3′. Human GAPDH sense primer: 5′-AGC CAC ATC GCT CAG ACA-3′; GAPDH antisense primer 5′-GCC CAA TAC GAC CAA ATC C-3′. Fluorescence data were acquired after the final extension step. A melt analysis was conducted for all products to determine the specificity of the amplification [24].

### 2.9. Small Interfering RNA

A specific double-stranded 21-nucleotide RNA sequence homologous (small interfering RNA (siRNA)) to the target gene was used to silence HO-1 expression. The computer software and Silencer™ siRNA construction kit from Ambion (Austin, TX, USA) designed and synthesized siRNA for HO-1 (sequences of the ribonucleotides were 5′-rGAC UGC GUU CCU GCU CAA CdTdT-3′ and 5′-rGUU GAG CAG GAA CGC AGU CdTdT-3′) and negative control number 1 siRNA. HO-1 protein inhibition was assessed by immunoblot analysis following transfection of cells with HO-1-siRNA. Briefly, cells were transiently transfected with 20 nM siRNA using 8 *μ*l of siPORT Amine (Ambion) [24, 25].

### 2.10. Statistical Analyses

Data were expressed as means ± standard deviation (SD). Statistical evaluation was performed using Student's *t*-test or one-way analysis of variance, followed by Dunnett's test. A *P* value of <0.05 was considered significant.

## 3. Results

### 3.1. Isolation and Characterization of Circulating Bone Marrow-Derived PACs

**Figure 1:**
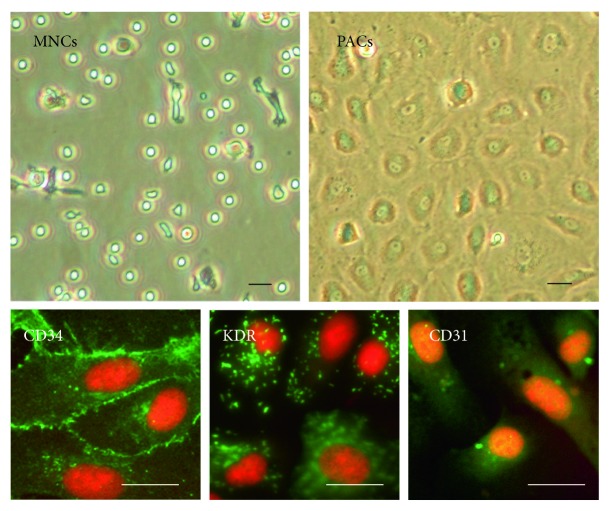
Characterization and morphology of PACs. MNCs were plated on fibronectin-coated plate on the first day (upper left). Late outgrowth PACs with cobblestone-like morphology were reseeded and grown to confluence (upper right). Immunofluorescence staining (green) of CD34, KDR, and CD31 for late outgrowth PACs. Cell nucleus was counterstained with propidium iodide (red). Scale  bar = 50 *μ*m.

Cells originated from peripheral blood MNCs of healthy volunteers. MNCs initially seeded on fibronectin-coated wells were round-shaped. Late outgrowth PACs with cobblestone-like morphology were grown to confluence (Figure 1).

Cell characterization was performed by fluorescent stain. CD34, KDR, and CD31 may be considered markers of late outgrowth PACs. CD34 and KDR double positive may be important markers of these cells in vitro (Figure 1) [8, 26]. Endothelial marker CD31 was also used for characterization of the outgrowth cells.

### 3.2. RYR Extract Is Toxic Only in High Concentrations

**Figure 2:**
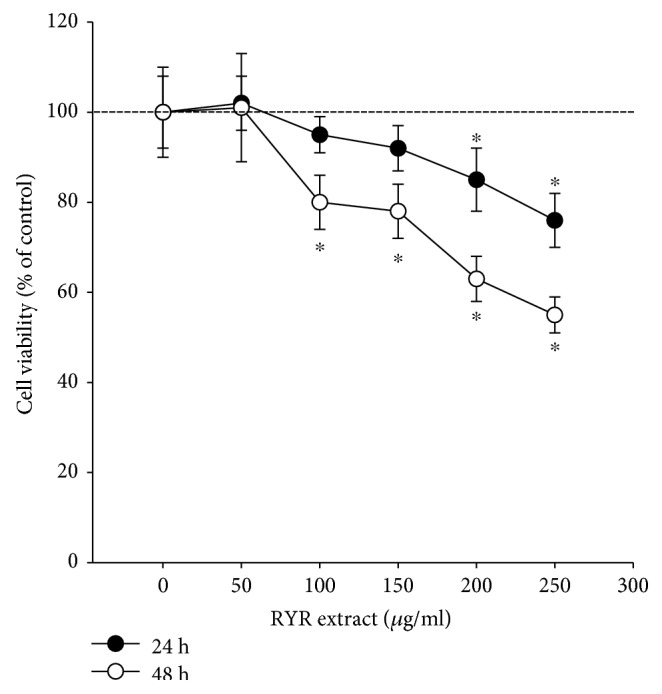
PAC viability after incubation with RYR extract for 24 h and 48 h is determined by MTT assay. Data are expressed as percentage (mean ± SD) of survival cells by the control group. The results are from six separate experiments, ^∗^*P* < 0.05 compared to that of the control group.

Incubation of PACs with 0–50 *μ*g/ml RYR extract for 24 h and 48 h did not result in cellular toxicity; however, high doses of RYR extract (≥200 *μ*g/ml for 24 h and ≥100 *μ*g/ml for 48 h) significantly reduced cell viability (Figure 2). These data indicate that the significant cytotoxic effects of RYR extract on PACs were found in high doses. Thus, the noncytotoxic doses of RYR extract (≤50 *μ*g/ml) in the following experiments were used to avoid potential interference of cell survival.

### 3.3. RYR Extract Induces Nrf-2 Activation and HO-1 Expression in PACs

**Figure 3:**
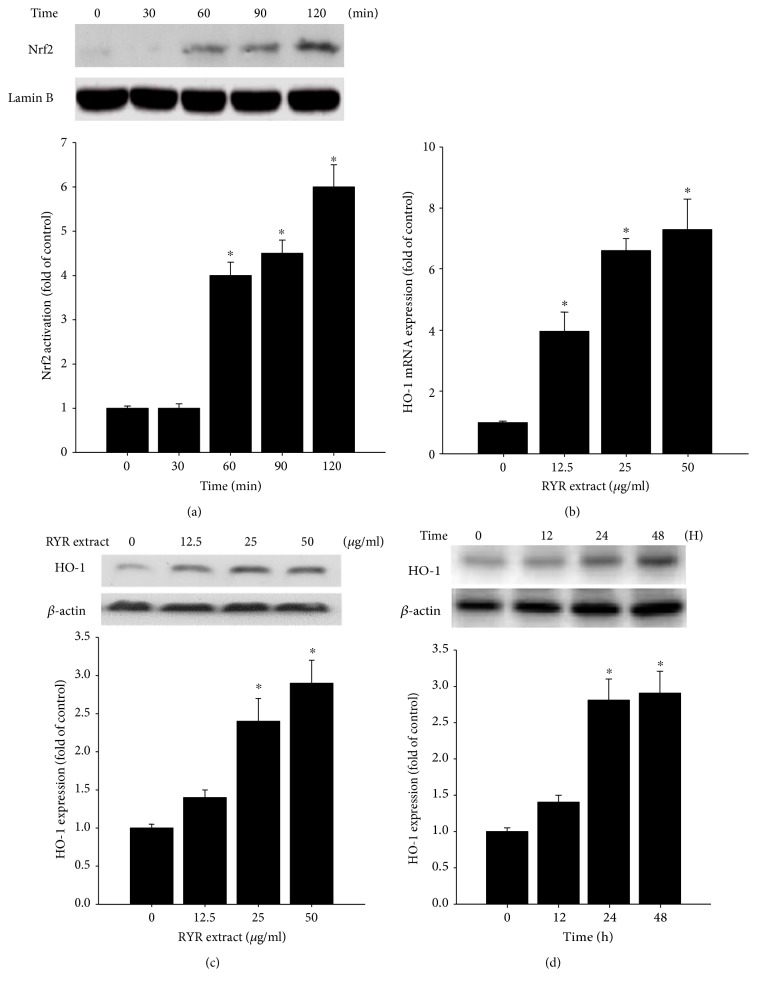
(a) RYR extract time-dependently induces Nrf2 nuclear translocation in PAC cells after RYR extract (50 *μ*M) incubation. (b) RYR extract dose-dependently induces HO-1 mRNA expression (12 h) in PACs. RYR extract (c) dose- and (d) time-dependently induces HO-1 protein expression in PACs. Data are expressed as mean ± SD of three independent experiments. ^∗^*P* < 0.05 compared with that of the medium alone control group.

We further tested the effects of RYR extract on Nrf2 signaling pathway and HO-1 expression in PACs. 50 *μ*g/ml RYR extract time-dependently induced Nrf2 nuclear translocation in PAC cells (Figure 3(a)). In addition, RYR extract (12.5, 25, and 50 *μ*g/ml) was added to culture medium with PACs, and real-time PCR and Western blot were performed for HO-1 mRNA (12 h) and protein (48 h) expressions, respectively. As shown in Figures 3(b) and 3(c), RYR extract increased HO-1 mRNA and protein expression in a dose-dependent manner. Moreover, 50 *μ*g/ml of RYR extract increased HO-1 protein expression in PACs in a time-dependent manner (12, 24, and 48 h) (Figure 3(d)).

### 3.4. RYR Extract Inhibits High-Glucose-Induced Senescence and Oxidative Stress

**Figure 4:**
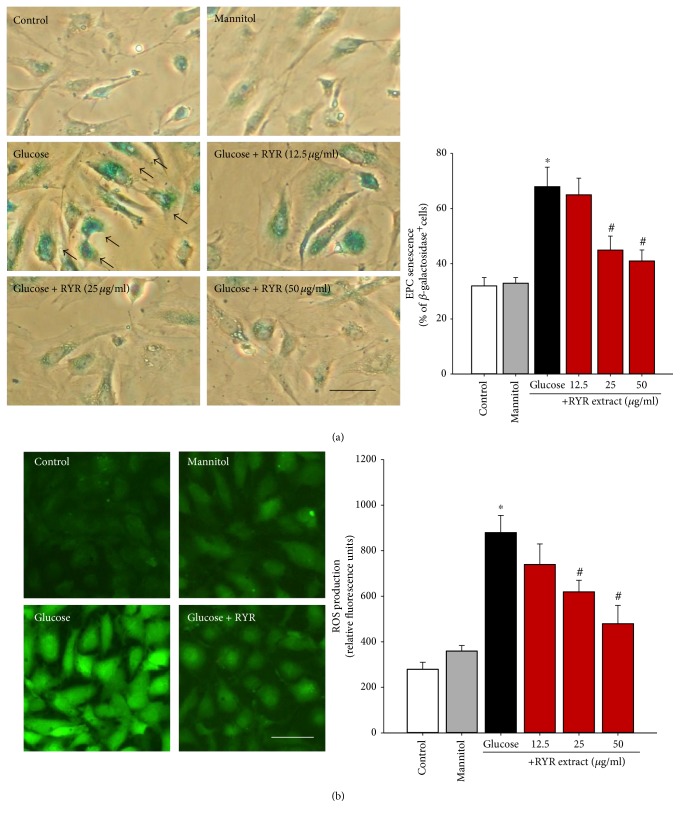
RYR extract dose-dependently inhibits (a) cell senescence and (b) ROS production in high-glucose-treated PACs. Arrows: strong blue-stained *β*-galactosidase-positive cells. Data are expressed as mean ± SD of three independent experiments. ^∗^*P* < 0.05 compared to that of the control group; ^#^*P* < 0.05 compared to that of the high-glucose-treated group.

Our previous study demonstrated that high-glucose-caused (30 mM) senescence and oxidative stress in PACs as compared with the control (5 mM of glucose) or osmotic control (extra 25 mM of mannitol) groups [8]. To investigate whether RYR extract inhibited senescence of PACs induced by high glucose, PACs were coincubated with high glucose (30 mM) and RYR extract (12.5–50 *μ*g/ml) for 48 h, and a *β*-galactosidase assay was performed. RYR extract had a dose-dependent effect to reduce senescence in high-glucose-treated PACs (Figure 4(a)).

In addition, to directly determine the effect of RYR extract on ROS generation, we analyzed the ROS level in high-glucose-treated PACs. As shown in Figure 4(b), treatment with high glucose for 48 h caused a higher increase of fluorescence compared with the control and mannitol groups. Coincubation of PACs with RYR extract inhibited high-glucose-induced ROS generation in a dose-dependent manner.

In addition, Table 1 shows that RYR extract treatment caused a significant increase of GSH content and GR activity but a significant decrease of thiobarbituric acid reactive substance (TBARS) content relative to high-glucose-treated PACs.

### 3.5. RYR Extract Inhibits High-Glucose-Induced Senescence and Oxidative Stress via HO-1

**Figure 5:**
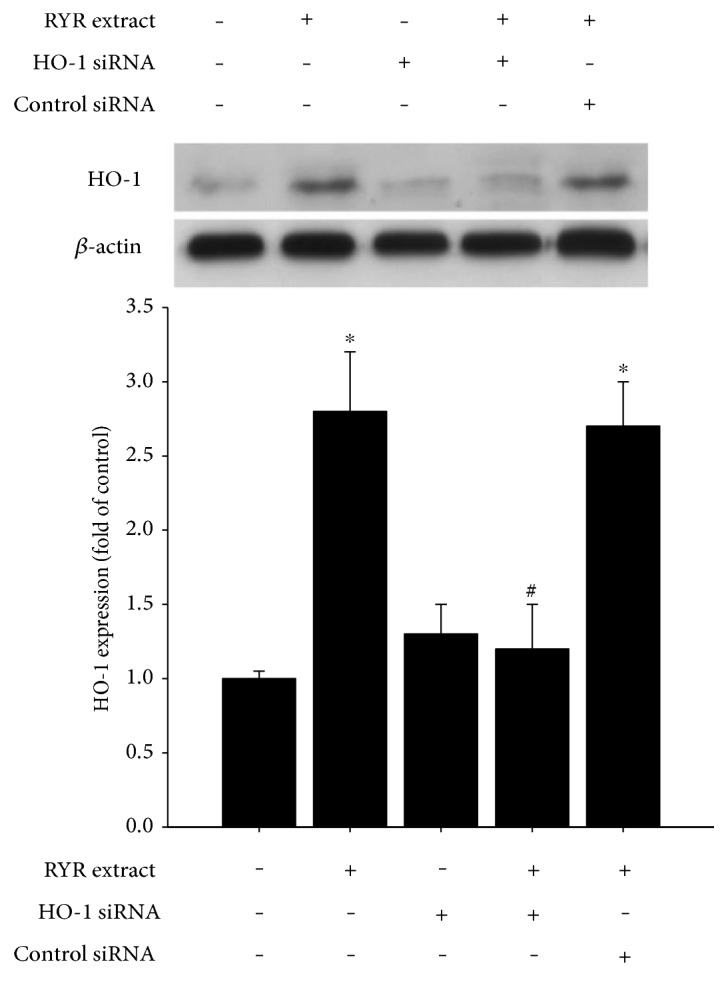
HO-1 siRNA inhibits RYR extract-induced HO-1 protein expression. PACs were transfected with HO-1 siRNA or control siRNA and then stimulated with RYR extract (50 *μ*g/ml). Cell lysates were subjected to Western blotting to determine levels of HO-1 and *β-*actin. Data are expressed as mean ± SD of three independent experiments. ^∗^*P* < 0.05 compared to that of the control group; ^#^*P* < 0.05 compared to that of the RYR extract-treated group.

**Figure 6:**
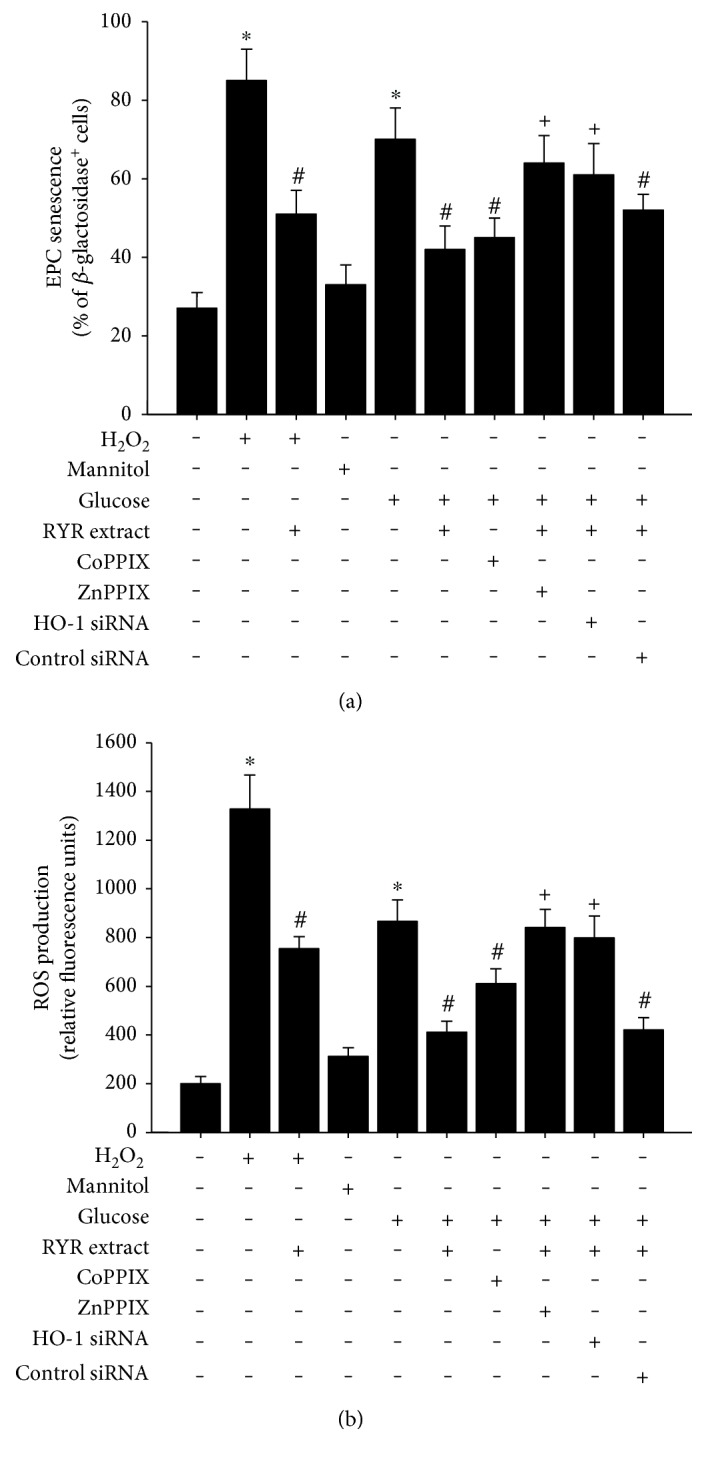
CoPPIX (10 *μ*M), ZnPPIX (10 *μ*M), and HO-1 siRNA modulate the inhibitory effect of RYR extract on high-glucose-caused (a) senescence and (b) oxidative stress in PACs. Oxidative stimulator H_2_O_2_ (50 *μ*M) was used to confirm the antioxidative effect of RYR extract. Data are expressed as mean ± SD of three independent experiments. ^∗^*P* < 0.05 compared to that of the control group; ^#^*P* < 0.05 compared to that of the H_2_O_2_ or high-glucose-treated group; ^+^*P* < 0.05 compared to that of the RYR extract and high-glucose-treated group.

HO-1 siRNA was used to confirm the effects of RYR extract mediated through HO-1.  Figure 5 shows a reduction of RYR extract-induced HO-1 by HO-1 siRNA. We further explored the effect of HO-1 on high-glucose-induced PAC senescence and oxidative stress. As shown in Figures 6(a) and 6(b), we found that the HO-1 inducer cobalt protoporphyrin (CoPPIX) also significantly decreased high-glucose-induced PAC senescence and oxidative stress, whereas the HO-1 enzyme inhibitor, zinc protoporphyrin IX (ZnPPIX), and HO-1 siRNA significantly reversed RYR extract-caused inhibition in high-glucose-treated PACs. Oxidative stimulator H_2_O_2_ (50 *μ*M) was also used to confirm the antioxidative effect of RYR extract.

## 4. Discussion

The present study showed, for the first time, that RYR extract attenuated high-glucose-induced senescence and oxidative stress of PACs. Our data also suggest that HO-1 activation may play a pivotal role in the anticellular senescence and antioxidative effects of RYR extract on PACs.

As the incidence of myocardial infarction and stroke increases as the population ages, there must be an increased focus on the fundamental processes and mechanisms of vascular aging. An advanced understanding of the molecular pathways leading to vascular aging may contribute to the design of therapeutic strategies to prevent vascular senescence. More recently, Paneni et al. reviewed the advances in the pathology of age-related vascular dysfunction including dysregulation of epigenetic modifications, inflammatory genes, and mechanisms of vascular calcification [27]. Oxidative stress contributes to the progression of endothelial-dysfunction-related clinical diseases through luminal narrowing in the brain (ischemic stroke), heart, and peripheral vessels [28]. Epidemiological studies indicate that RYR consumption is associated with reduced coronary heart disease risk [29, 30]. In vitro investigations have indicated that RYR can inhibit several key events of the atherogenic process, such as vascular smooth muscle and endothelial cell dysfunction by redox-sensitive mechanisms [15, 16, 31]. RYR contains chemicals that are similar to prescription statin medications including monacolin K (the same structure as the drug lovastatin). Statin-mediated HO-1 induction has been shown to occur in vascular smooth muscle cells [32, 33], endothelial cells [34–40], macrophages [41, 42], neurons [43–46], liver cells [47, 48], and pulmonary cells [49, 50]. In this study, we showed, for the first time, that RYR extract induces HO-1 activation in bone-marrow-derived PACs. HO-1 expression is mediated through accumulation of Nrf2 in the nucleus. We also found that RYR extract induces Nrf2 nuclear translocation in PACs. Moreover, the antioxidant properties of RYR extract were further reported to protect against cellular senescence by inhibiting high-glucose-induced oxidative stress in PACs via HO-1 induction. Thus, our results elucidated the relationship with hyperglycemia, oxidative stress, and endothelial dysfunction, regulation of atheroprotective genes HO-1, and how the regulation of these activities by RYR can lead to the prevention of diabetes-related vascular complications.

During atherogenesis, hyperglycemia-mediated chronic oxidative stress plays an important role in PAC dysfunction [51]. In the present study, RYR extract induced HO-1 expression in PACs in a dose- and time-dependent manner. Consistent with the present results, various vascular protective agents such as atorvastatin [52], estradiol [53], and oleuropein/oleacein (phenolic compounds from olive oil) [54] also increase HO-1 and display anti-inflammatory effects in EPCs. All these data suggest that RYR is a strong inducer of HO-1, and such induction may be independent of various vascular protective agents.

ROS have been implicated in the pathogenesis of most stages of atherosclerosis [11, 55, 56]. ROS, especially hydrogen peroxide and superoxide, are important intracellular signaling molecules in cells. ROS participate in the growth and death of PACs; these events play crucial roles in cardiovascular diseases, suggesting that the sources of ROS and the intracellular signaling pathways may be important therapeutic targets [57]. Evidence has shown that ROS influences cellular processes in vascular remodeling by activating various intracellular signaling cascades [57]. Our previous in vitro study demonstrated the activity of RYR extract on the radical-scavenging abilities of the probe-based ultraweak chemiluminescence technique and showed that RYR exhibited major radical-scavenging abilities on superoxide and hydroxyl radicals [16]. The present study further provides direct evidence that RYR extract maintained GSH amounts and upregulated GR activity in high-glucose-stressed PACs resulting in decreased TBARS, suggesting that RYR could maintain the intracellular antioxidant concentrations in biological systems. It should be further examined whether RYR upregulates other GSH-related enzymes, such as glutamate cysteine ligase and glutathione peroxidase, which catalyze GSH biosynthesis.

Our study has limitations. The composition of the various compounds from the RYR extract, in particular those that might be responsible for the protective effects in the RYR mixture and may specifically induce HO-1, is not clearly defined in this manuscript; only the crude extract of RYR was studied. Moreover, the molecular mechanism underlying HO-1 induction by RYR is unclear. For example, it is unknown if nuclear factor erythroid 2-related factor 2, a major transcriptional regulator of HO-1 expression [58], or the HO products carbon monoxide and bilirubin [59] were involved in the protective effects of RYR on PACs. Finally, the measurement of some direct inflammation markers could provide more evidence on the anti-inflammatory role of HO-1 after RYR treatment. It is important to explore the effective compounds and mechanisms of RYR for further direction in the field of agricultural product research.

## 5. Conclusions

The present study demonstrated that RYR extract induced HO-1 expression in PACs in a dose- and time-dependent manner. RYR extract inhibited high-glucose-induced *β*-galactosidase activation and reduced high-glucose-induced oxidative stress in PACs in a dose-dependent manner. HO-1 expression might play a pivotal role in the atheroprotective effects of RYR on PACs. Thus, RYR may fulfill the definition of a pharmacological preconditioning agent for preventing cerebrovascular and cardiovascular diseases.

## Figures and Tables

**Table 1:** The GSH, GR, and TBARS levels in PACs.

	Control	Mannitol	Glucose	Glucose + RYR extract
GSH (nmol/mg protein)	52.6 ± 8.3	46.3 ± 3.9	29.3 ± 2.6^∗^	48.5 ± 5.5^#^
GR (unit/mg protein)	1.8 ± 0.2	1.6 ± 0.2	1.1 ± 0.1^∗^	1.6 ± 0.1^#^
TBARS (nmol/mg protein)	2.8 ± 0.5	3.2 ± 0.6	8.2 ± 1.1^∗^	3.6 ± 1.9^#^

^∗^ *P* < 0.05 compared to that of the control group; ^#^*P* < 0.05 compared to that of the glucose group.
